# Predicting global potential distribution of *Peromyscopsylla hesperomys* and *Orchopeas sexdentatus* and risk assessment for invading China under climate change

**DOI:** 10.3389/fpubh.2022.1018327

**Published:** 2023-01-05

**Authors:** Hongyun Li, Ying Liang, Li Dong, Cancan Li, Lu Zhang, Bin Wang, Delong Ma, Qunzheng Mu, Jun Wang, Haifeng Hou, Qiyong Liu

**Affiliations:** ^1^School of Public Health, Shandong First Medical University and Shandong Academy of Medical Sciences, Jinan, China; ^2^State Key Laboratory of Infectious Disease Prevention and Control, National Institute for Communicable Disease Control and Prevention, Chinese Center for Disease Control and Prevention, Beijing, China; ^3^School of Public Health, Cheeloo College of Medicine, Shandong University, Jinan, China; ^4^Beijing Key Laboratory of Clinical Epidemiology, School of Public Health, Capital Medical University, Beijing, China; ^5^School of Public Health, Jiamusi University, Jiamusi, China; ^6^Department of Infectious Diseases, Shizhong Center for Disease Control and Prevention, Jinan, China; ^7^School of Public Health and The Second Affiliated Hospital of Shandong First Medical University, Taian, China; ^8^Shandong University Climate Change and Health Center, School of Public Health, Shandong University, Jinan, China

**Keywords:** global distribution, climate change, potential suitable areas, risk assessment, plague vector fleas

## Abstract

**Background:**

*Peromyscopsylla hesperomys* and *Orchopeas sexdentatus* are regarded to be representative plague vectors in the United States. The incidence of plague is rising globally, possibly due to climate change and environmental damage. Environmental factors such as temperature and precipitation have a significant impact on the temporal and spatial distribution of plague vectors.

**Methods:**

Maximum entropy models (MaxEnt) were utilized to predict the distributions of these two fleas and their trends into the future. The main environmental factors influencing the distribution of these two fleas were analyzed. A risk assessment system was constructed to calculate the invasion risk values of the species.

**Results:**

Temperature has a significant effect on the distribution of the potentially suitable areas for *P. hesperomys* and *O. sexdentatus*. They have the potential to survive in suitable areas of China in the future. The risk assessment system indicated that the risk level for the invasion of these two species into China was moderate.

**Conclusion:**

In order to achieve early detection, early interception, and early management, China should perfect its monitoring infrastructure and develop scientific prevention and control strategies to prevent the invasion of foreign flea vectors.

## Introduction

Globally, flea-borne infectious diseases, for instance, plague, are creating a comeback and their incidence is on the rise, which may be related to global climate change and environmental damage (1). Climate change is defined as a modification of temperature and weather patterns over a long-term cycle. Global warming is an incontrovertible fact, with the temperatures of global lands and oceans increasing by an average of 0.89°C (0.69–1.08°C) from 1901 to 2012 (2).

The World Health Organization estimates that the number of excess deaths attributable to global climate change will reach 250,000 per year in 2030–2050. Moreover, global warming has vital implications for vector-borne infectious diseases: First, the temporal and geographic distribution of vector organisms is susceptible to climatic and environmental factors, and a tiny increase in temperature could cause obvious changes in the population size and distribution range of vector organisms. Second, climatic factors influence the reproduction of pathogenic microorganisms in vector organisms considerably (3). Due to future climate change, the risk of plague in the temperate zone might grow significantly (4). The occurrence of extreme weather events and natural disasters increases the likelihood of large-scale outbreaks and epidemics of plague (5).

Fleas are vectors of the plague bacterium (6). They transmit plague *via* the formation of *Yersinia pestis* bacterial embolus, and the speed of this process is regulated by temperature (7, 8). Climate change impacts the fluctuations of the flea population *via* variations in temperature and humidity and elevates the risk of biological invasion (9). The risk assessment of biological invasion includes invasion risk, colonization risk, spread risk, and damage consequences. The premise of colonization is that the alien species has a suitable zone. The ecological niche model (ENM) provides an important quantitative analysis tool for predicting the distribution of specie's suitable zones, which uses distribution data of known species and relevant environmental variables to determine the ecological requirements of species based on the constructed model and projects the results into different spatial and temporal dimensions (10). The maximum entropy (MaxEnt) model has better predictive performance than other ecological niche models (11), and it can predict the future distribution of species' suitable zones, while others cannot, therefore, we chose the MaxEnt model. The model is an algorithm for predicting species' suitable areas based on the probability distribution and has the advantages of wide application, high prediction accuracy, and low requirements for sample content (12–16).

In total, 464 human plague cases were reported in the United States (U.S.) from 1950 to 2009 (17). In the U.S., most human cases are normally associated with flea bites (18). Data indicated that the U.S. is the first exporter to China and the third importer to China in 2020. With the development of global economic integration, the increasing flow of cargo between China and the U.S. has added the risk of the entry of plague vector fleas because fleas can be attached to host, clothing, and textiles imported by vehicles/ships, which has the potential to cause an epidemic of plague in China. Therefore, it is significant to study the global distribution of U.S. plague vector fleas and the risk of them invading China by using the MaxEnt model to prevent the introduction of plague into China.

In this research, *Peromyscopsylla hesperomys* (*P. hesperomys*) and *Orchopeas sexdentatus* (*O. sexdentatus*) were selected for the predicting suitable areas and the invasion risk assessment.

## Materials and methods

### Materials

#### Sources and screening of occurrence data

Literature searches were conducted using China National Knowledge Infrastructure (CNKI), PubMed, Web of Science, Embase, and Google Scholar. Global Invasive Species Database (GISD), Invasive Species Compendium (ISC), European and Mediterranean Plant Protection Organization (EPPO), World Health Organization (WHO), and the U.S. Centers for Disease Control and Prevention (CDC) were used to screen for fleas with invasive risk and positive for plague *Yersinia pestis* (19). Two representative U.S. fleas: *P. hesperomys* and *O. sexdentatus*, were identified as vectors of plague and selected for the study.

**Figure 1:**
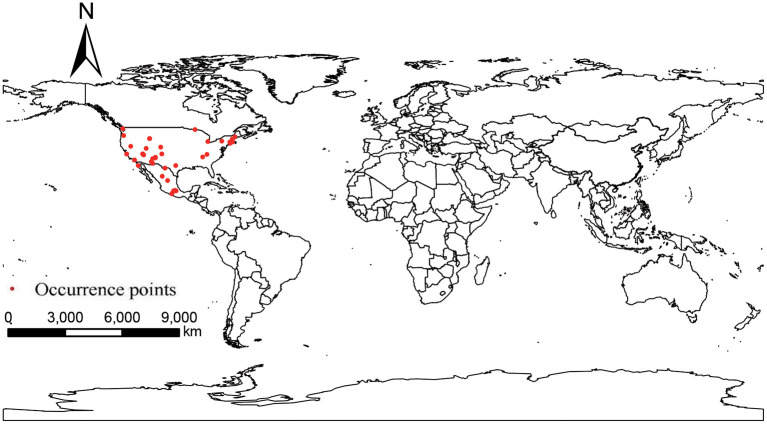
Global occurrence of *P. hesperomys*.

**Figure 2:**
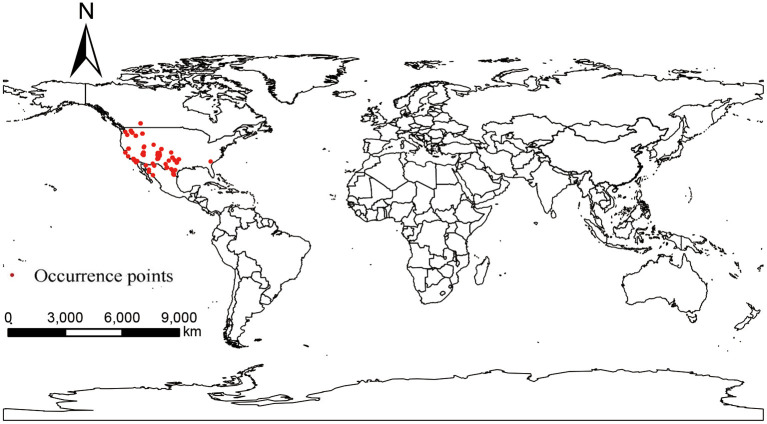
Global occurrence of *O. sexdentatus*.

Occurrence points of *P. hesperomys* and *O. sexdentatus* were obtained from the Global Biodiversity Information Facility (GBIF) (https://www.gbif.org; accessed on 5 April 2022). The accuracy of the coordinate points was checked. When the species distribution data were too dense in a particular area, ENMTools software (https://www.activestate.com/products/perl/downloads; accessed on 12 April 2022) is used to keep only one distribution point within the same raster to avoid result overfitting (20), and we obtained 39 occurrence points for *P. hesperomys* (Figure 1) and 65 for *O. sexdentatus* (Figure 2).

### Screening of environmental data

Environmental variables, acquired from the near-current climate scenario (1970–2000) and future climate scenarios (2021–2040, 2041–2060, 2061–2080, and 2081–2100), were collected from WorldClim (version 2.1, http://www.worldclim.org; accessed on 5 April 2022) by a spatial resolution of 5 arc-min, containing bioclimatic variables (Bio1–Bio19), monthly maximum temperature (Tmax1–Tmax12), monthly minimum temperature (Tmin1–Tmin12), monthly precipitation (Prec1–Prec12), and elevation (Ele). They were processed by the BCC-CSM2-MR (Medium-Resolution Beijing Climate Center Climate System Model version 2) under four different socio-economic models driven by CO_2_: shared socio-economic pathways (SSPs) 126, 245, 370, and 585 (21).

To avoid the overfitting of environmental variables, Maxent was used to get the contribution rate of environmental variables. R software was used (version 4.1.0 https://www.r-project.org; accessed on 15 February 2022) to conduct Pearson's correlation analysis on environmental variables. If the absolute value of the correlation coefficient between two variables was >0.8, the variable with a smaller contribution was removed. If the absolute value of the correlation coefficient was < 0.8, both variables were retained. In the end, 10 variables were filtered as predictors of *P. hesperomys*, and 8 variables were filtered as predictors of *O. sexdentatus* (Table 1).

**Table 1:** Percentage contributions of environmental variables in Maxent for *P. hesperomys* and *O. sexdentatus*.

**Abbreviation**	**Variables**	***P. hesperomys* contribution (%)**	***O. sexdentatus* contribution (%)**
Tmin11	Minimum temperature of November	46.2	
Tmin1	Minimum temperature of January	19.8	18.4
Bio2	Mean diurnal range (mean of monthly [max temp—min temp)]	15.4	24.1
Bio14	Precipitation of the driest month	4.1	7.2
Bio3	Isothermality (Bio2/Bio7 × 100) (%)	4.1	29.7
Ele	Elevation	3.5	
Tmin12	Minimum temperature of December	3.4	
Bio8	Mean temperature of wettest quarter	2.5	
Prec1	Precipitation in January	2.4	11.1
Prec9	Precipitation in September	2.2	3.5
Prec7	Precipitation in July		4.2
Bio9	Mean temperature of driest quarter		1.8

Bio7, temperature annual range (Bio5–Bio6); Bio5, max temperature of the warmest month; Bio6, min temperature of the coldest month.

### Map data

The China map (scale: 1:4,000,000) is from the National Geomatics Center of China (http://www.ngcc.cn/ngcc; accessed on 12 April 2022), and the world map (scale: 1:10,000,000) is from the Natural Earth (https://www.naturalearthdata.com/downloads; accessed on 12 April 2022).

## Methods

### Maximum entropy model and parameter optimization

MaxEnt software (version 3.4.1, https://biodiversityinformatics.amnh.org; accessed on 5 April 2022) was used to predict the suitable areas for *P. hesperomys* and *O. sexdentatus* under near-current and future climate scenario model. The training and testing data sets were randomly generated, with 75% of the distribution points for training and 25% for testing.

The regularization multiplier (RM) and feature combination (FC) affected the predictive performance and accuracy of the MaxEnt model (22). R software was utilized to calculate RM and FC to optimize the MaxEnt model parameters (23). The RM parameter was usually set to eight levels: 0.5, 1, 1.5, 2, 2.5, 3, 3.5, and 4. The FC levels usually set five characteristic parameters: automatic linear (L), quadratic (Q), fragmentation (hinge, H), product (P), and threshold (T) to obtain eight features (L, LQ, LQP, QHP, LQH, LQHP, QHPT, and LQHPT). We used the R software package “ENMeval” to obtain the best model by Akaike's information criterion (AIC). When deltaAICc is equal to 0, the model is deemed to be the most appropriate (24). The RM and FC in this study were LQP and 1 in *P. hesperomys*, and QHP and 1 in *O. sexdentatus*, respectively. Jackknife tests were used to assess contribution rates of environmental variables, and the receiver operating characteristic (ROC) curve was used to evaluate the performance of the prediction model. The area under the curve (AUC) value, which can judge the model accuracy, is defined as the area under the ROC curve. The judgment standard: AUC (0.5–0.7), low predictive; AUC (0.7–0.9), middle predictive; AUC (0.9–1), highly predictive.

### Classification of the suitable areas

ArcGIS software (version 10.5) was purchased by the Department of Vector Biological and Control of the National Institute for Communicable Disease Control and Prevention, Chinese Center for Disease Control and Prevention (ICDC). Based on the natural break points, the probability of presence was classified into four categories: unsuitable area, low suitable, middle suitable, and high suitable, using ArcGIS's reclassification tool with complementary cloglog values (25).

### Risk assessment of *P. hesperomys* and *O. sexdentatus* invading China

In this research, a multi-indicator invasion risk assessment system was designed by referring to the previous invasion risk assessment index system (26, 27). An assessment index system of the invasion risk, colonization risk, spread risk, and damage consequences was conducted (Supplementary Table S1). Experts in the fields of biology, ecology, environmental science, and public health were invited to score each research index of the assessed species, and the assessment results were used to calculate the comprehensive invasion risk (R) value of the species as the risk for them invading China.

Based on the general process of foreign biological invasion (i.e., entry, colonization, spread, and damage), risk factors that affect biological invasion are divided into three categories, including the possibility of entry (*P*_1_), the possibility of colonization and spread (*P*_2_), and the damage consequences (*P*_3_). Each assessment factor is quantitatively or qualitatively described by specific elements.

Based on fuzzy mathematical principles, a uniform calculation was performed to classify each reference indicator into five grades according to the risk level, and a range was set for each level in the order of none [0], weak (0 < x ≤ 0.25), moderate (0.25 < x ≤ 0.5), strong (0.5 < x ≤ 0.75), and extremely strong (0.75 < x ≤ 1). Scores of quantitative indicators are assigned values based on specific situations, and qualitative indicators are scored by experts based on their professional knowledge and research experience. The R-value is calculated by *P*_1_, *P*_2_, and *P*_3_, which are logically related to each other. According to the multiplicative principle, the calculated formula is as follows: *R* = $\sqrt[{3}]{P_{1}\times P_{2}\times P_{3}}$.

The calculated risk value ranged from 0 to 1, which was classified as extremely low risk (0.01–0.05), low risk (0.05–0.3), moderate risk (0.3–0.7), and high risk (0.7–1).

The weight coefficients of the indicators were determined in accordance with the multiplication principle.

The entry possibility calculated formula is as follows:


$$\begin{array}{l} P_{1}\;=\;\sqrt[{2}]{P_{11}\times P_{12}}\\ P_{11}\;=\;{0.5\times \;P}_{111}+0.3\;P_{112}+\;0.2\;P_{113}\\ P_{12}\;=\;{0.5\times P}_{121}+\;0.5\;P_{122} \end{array}$$

Colonization and spread possibility calculated formulas are as follows:


$$\begin{array}{l} P_{2}\;=\;\sqrt[{5}]{P_{21}\times P_{22}\times P_{23}\times P_{24}\times P_{25}}\\ P_{21}\;=\;{0.6\times P}_{211}+0.2\;P_{212}+\;0.2\;P_{213}\\ P_{22}\;=\;{0.4\times P}_{221}+0.2\;P_{222}+\;0.2\;P_{223}\\ P_{24}\;=\;{0.5\times P}_{241}+\;0.5\;P_{242}\\ P_{25}\;=\;{0.2\times P}_{251}+0.3\;P_{252}+\;0.5\;P_{253} \end{array}$$

The damage consequences calculated formula is as follows:


$$\begin{array}{l} P_{3}\;=\;{0.6\times P}_{31}+\;0.4\;P_{32}\\ P_{31}\;=\;{0.3\times P}_{311}+0.3\;P_{312}+\;0.4\;P_{313}\\ P_{32}\;=\;{0.3\times P}_{321}+0.2\;P_{322}+{0.4\times P}_{323}+\;0.1\;P_{324} \end{array}$$

## Results

### Global suitable areas under near-current climate scenario models

**Figure 3:**
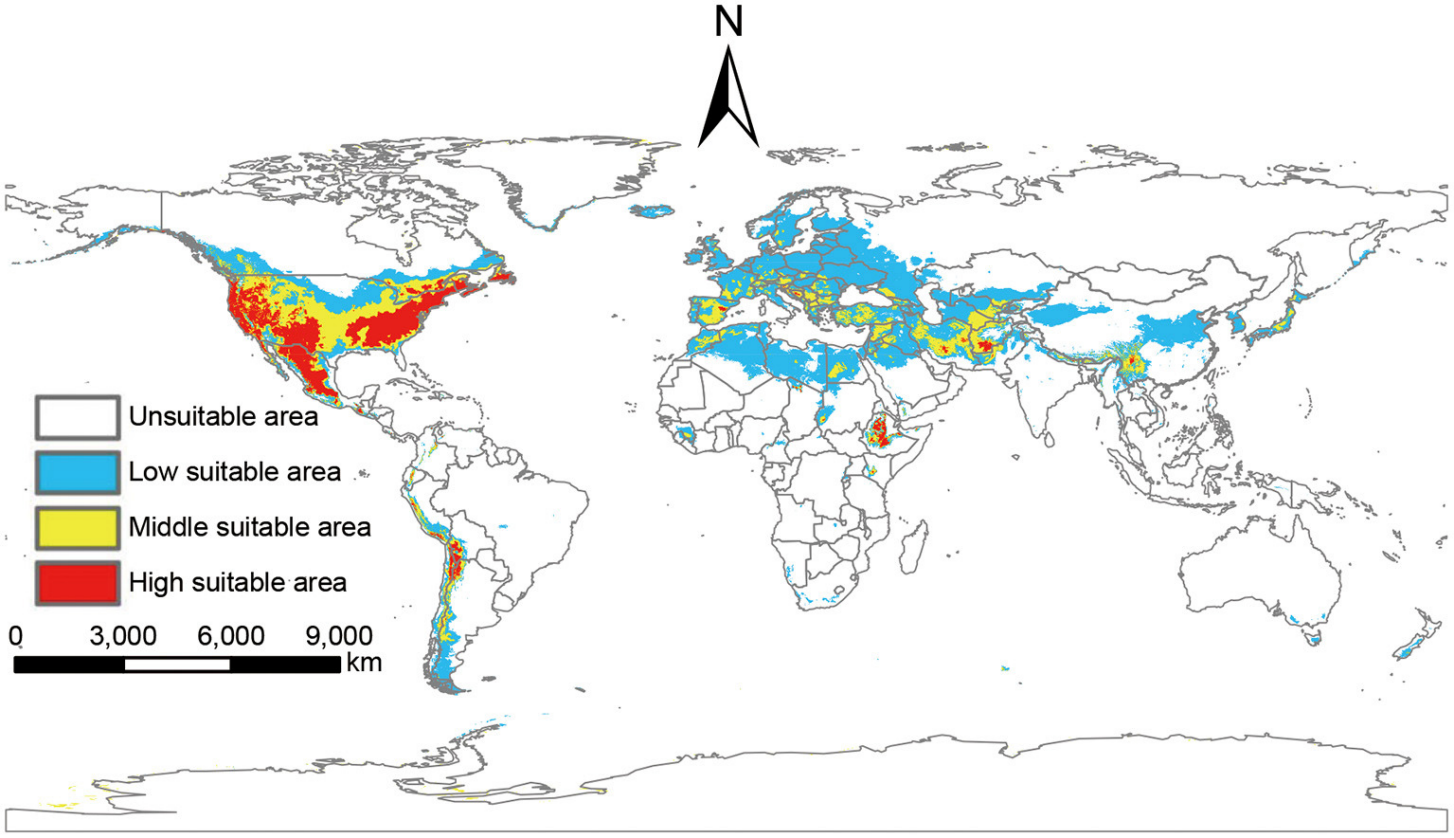
Suitable areas of *P. hesperomys* under near-current climate conditions.

Based on the output of MaxEnt, the global suitable areas of *P. hesperomys* under the near-current climate scenario were classified as unsuitable areas, low suitable areas, middle suitable areas, and high suitable areas. The suitable areas are mainly on the west coast of Canada, most of the states in the U.S. mainland and Alaska state, Mexico, and countries along the Andes Mountains. In addition, suitable areas are distributed in European including western Russia, North African countries (Libya, Algeria, Egypt, Morocco), west and Central Asian countries (Iraq, Iran, Afghanistan), and east Asia countries (China, Japan, and Korean Peninsula). The total global area of the suitable areas is 25.11 × 10^6^ km^2^ (Figure 3).

**Figure 4:**
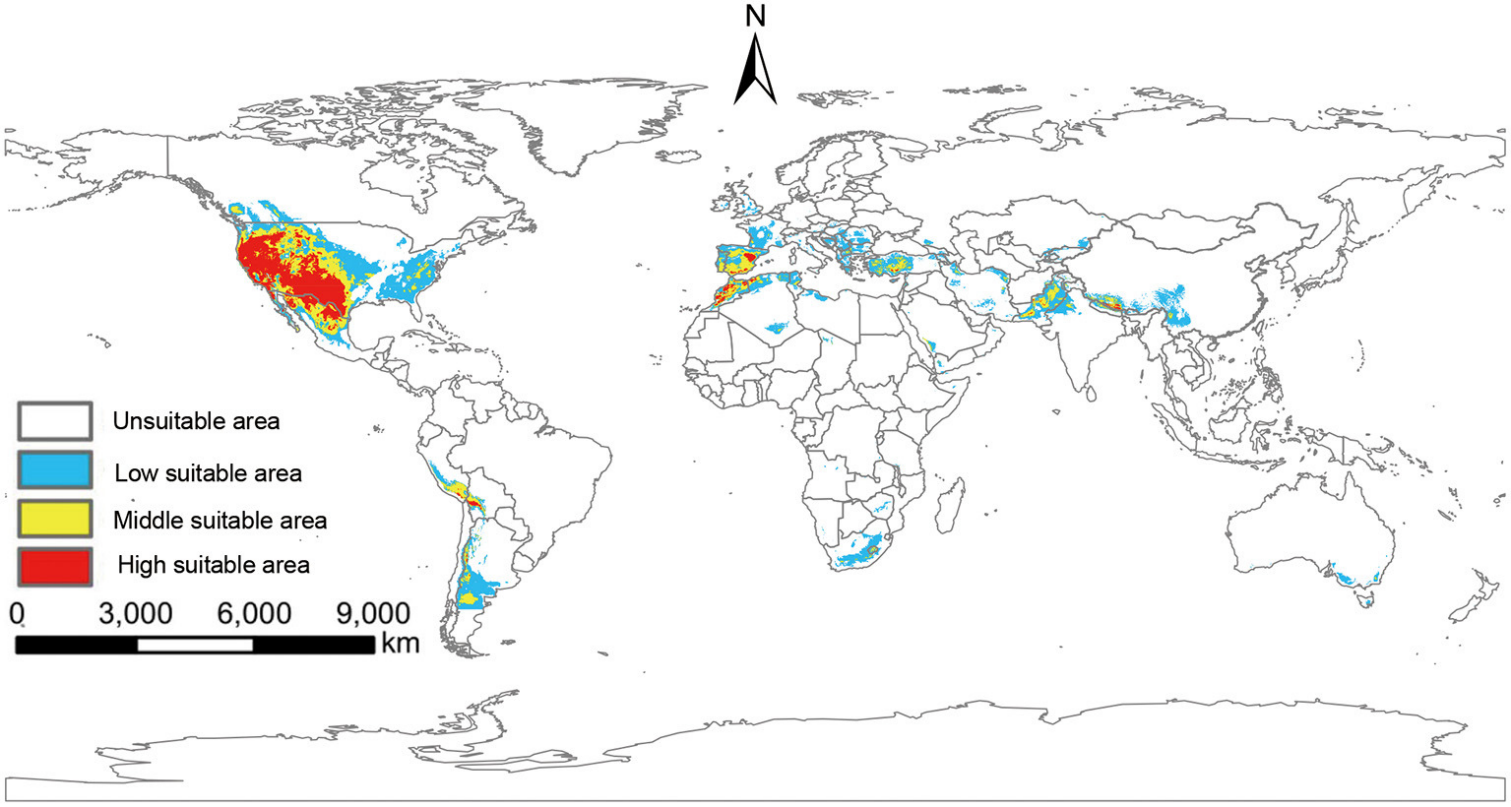
Suitable areas of *O. sexdentatus* under near-current climate conditions.

The potentially suitable areas for *O. sexdentatus* under near-current climate scenarios were mainly located in the west coast and Midwest of the U.S., the Southeastern U.S., northern Mexico, and southern Argentina in the Americas. In addition, the suitable areas for *O. sexdentatus* are located in Europe (Spain, Portugal, France, the United Kingdom, and Central and Eastern European countries), Africa (South Africa and the Mediterranean coastal areas countries), and Asia (Pakistan, Saudi Arabia, Iran, and China and other Central Asian countries). The total area of the global suitable areas is 9.11 × 10^6^ km^2^ (Figure 4).

### The suitable areas in China under future climate scenario models

**Figure 5:**
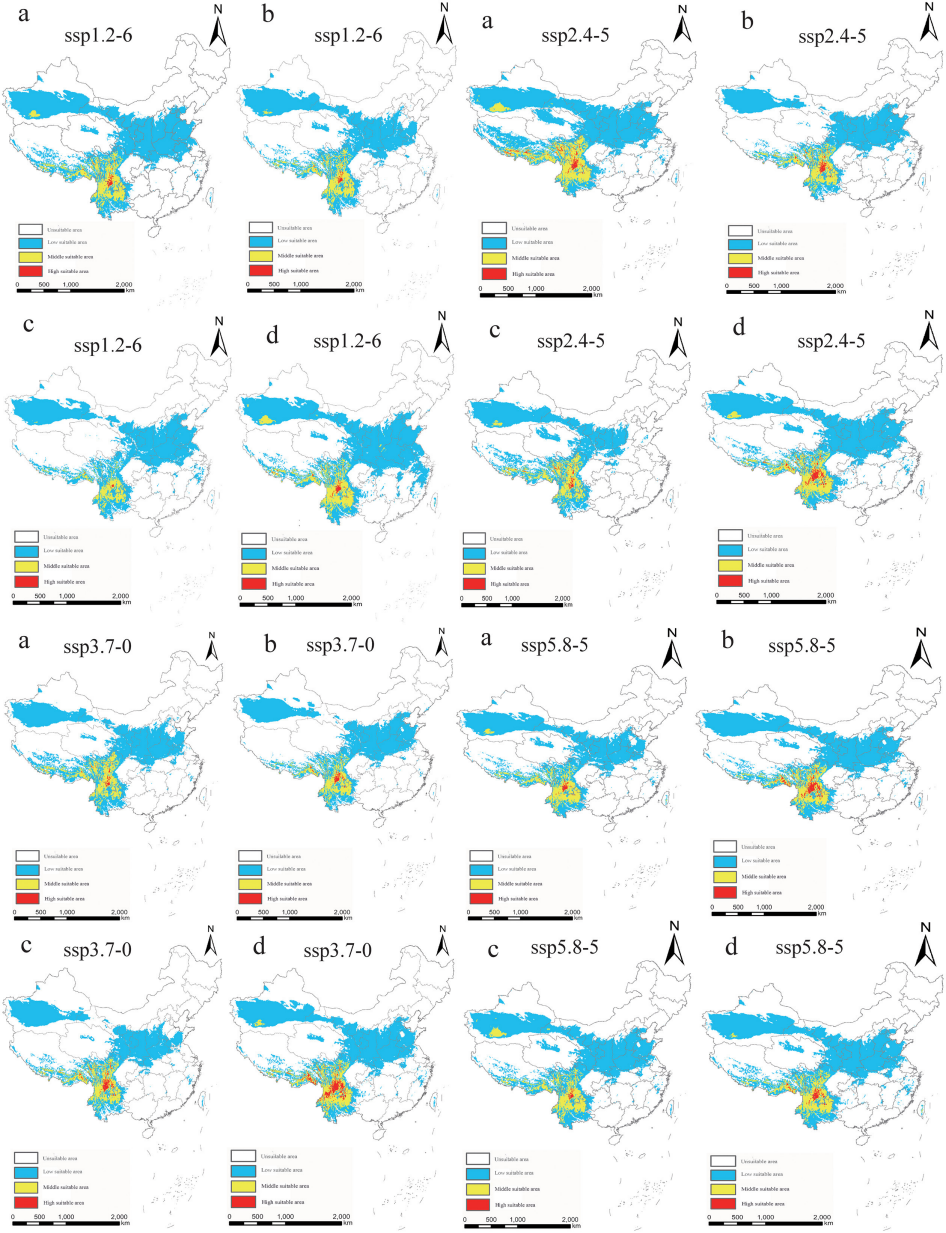
Suitable areas in China for *P. hesperomys* under different future climate scenario models (a: 2021–2040, b: 2041–2060, c: 2061–2080, and d: 2081–2100).

**Figure 6:**
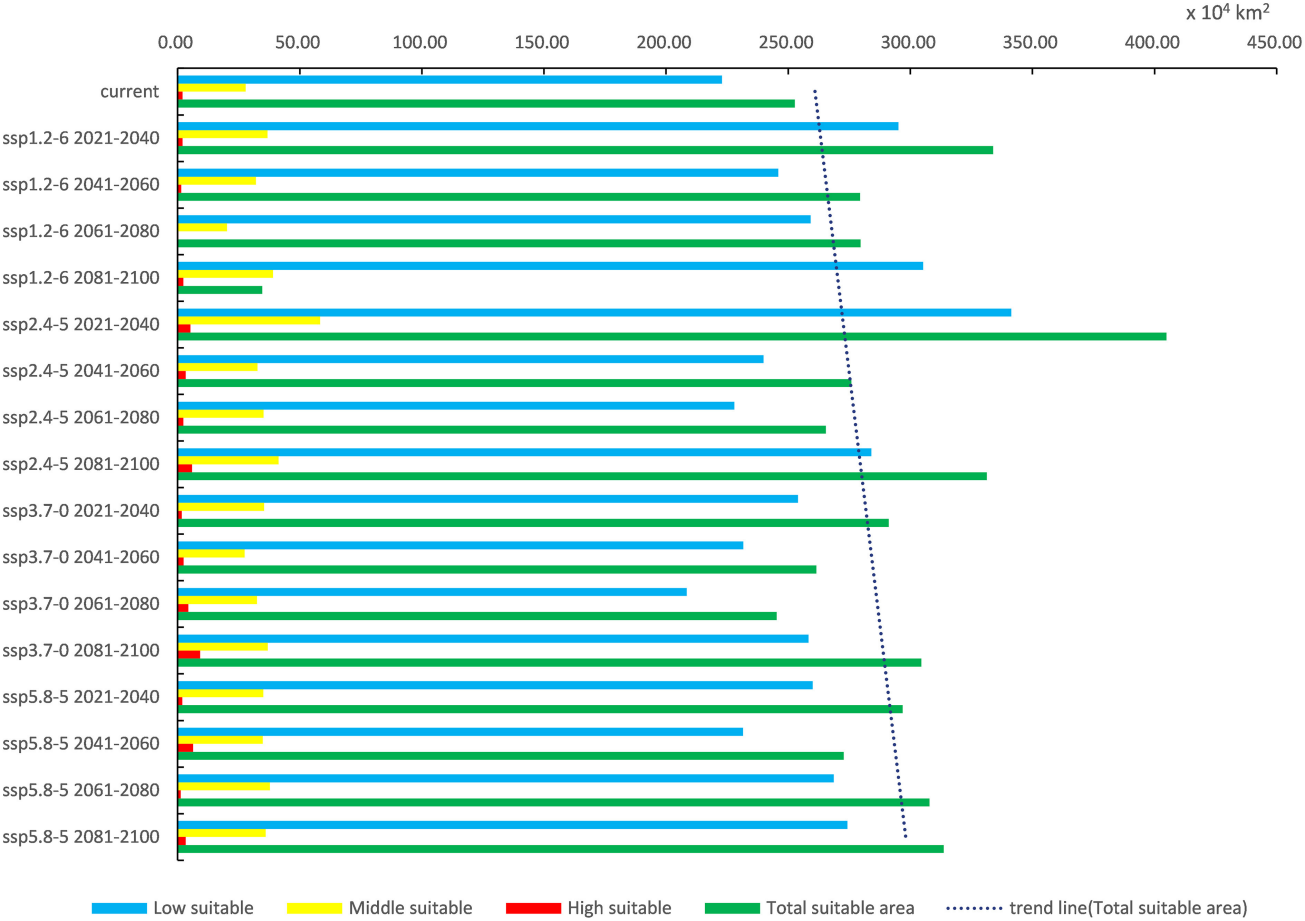
Variation trend of suitable areas for *P. hesperomys* in China under current and future climate scenarios.

Under each future climate scenario model, the potentially suitable areas for *P. hesperomys* in China are distributed in southern Tibet, Xinjiang, Yunnan, and western Sichuan. The low suitable area is mainly distributed in the Yellow River basin provinces of Qinghai, Sichuan, Ningxia, Inner Mongolia, Shaanxi, Shanxi, Henan, and Shandong and to a lesser extent in Anhui, Jiangsu, Zhejiang, Hubei, Beijing, Tianjin, and Hebei (Figure 5). As shown in Table 2, under ssp2.4-5 scenario models from 2021 to 2040, the suitable area for *P. hesperomys* has the largest range in China (404.94 × 10^4^ km^2^). The area potentially suitable for *P. hesperomys* under each climate scenario reveals an overall increasing trend in the future (Figure 6).

**Table 2:** Suitable areas of *P. hesperomys* in China under current and future climate scenarios ( × 10^4^ km^2^).

**Climate scenarios**	**Period**	**Low suitable**	**Middle suitable**	**High suitable**	**Total area**	**Area change**	**Area change ratio (%)**
Current	1970–2000	222.92	27.86	2.00	252.77	0.00	
ssp1.2–6	2021–2040	295.12	36.82	1.95	333.89	81.12	32.10
	2041–2060	245.91	32.06	1.42	279.39	26.62	10.53
	2061–2080	259.19	20.16	0.30	279.65	26.88	10.63
	2081–2100	305.29	39.10	2.31	346.70	93.93	37.16
ssp2.4–5	2021–2040	341.39	58.30	5.25	404.94	152.17	60.20
	2041–2060	239.90	32.68	3.26	275.84	23.07	9.13
	2061–2080	227.94	35.16	2.34	265.44	12.67	5.01
	2081–2100	284.10	41.36	5.86	331.32	78.55	31.08
ssp3.7–0	2021–2040	254.06	35.40	1.72	291.18	38.41	15.20
	2041–2060	231.66	27.41	2.46	261.53	8.76	3.47
	2061–2080	208.46	32.49	4.38	245.33	−7.44	−2.94
	2081–2100	258.37	36.87	9.24	304.48	51.71	20.46
ssp5.8–5	2021–2040	260.00	35.04	1.88	296.92	44.15	17.47
	2041–2060	231.54	34.86	6.35	272.75	19.98	7.90
	2061–2080	268.70	37.81	1.30	307.81	55.04	21.77
	2081–2100	274.29	36.01	3.33	313.63	60.86	24.08

**Figure 7:**
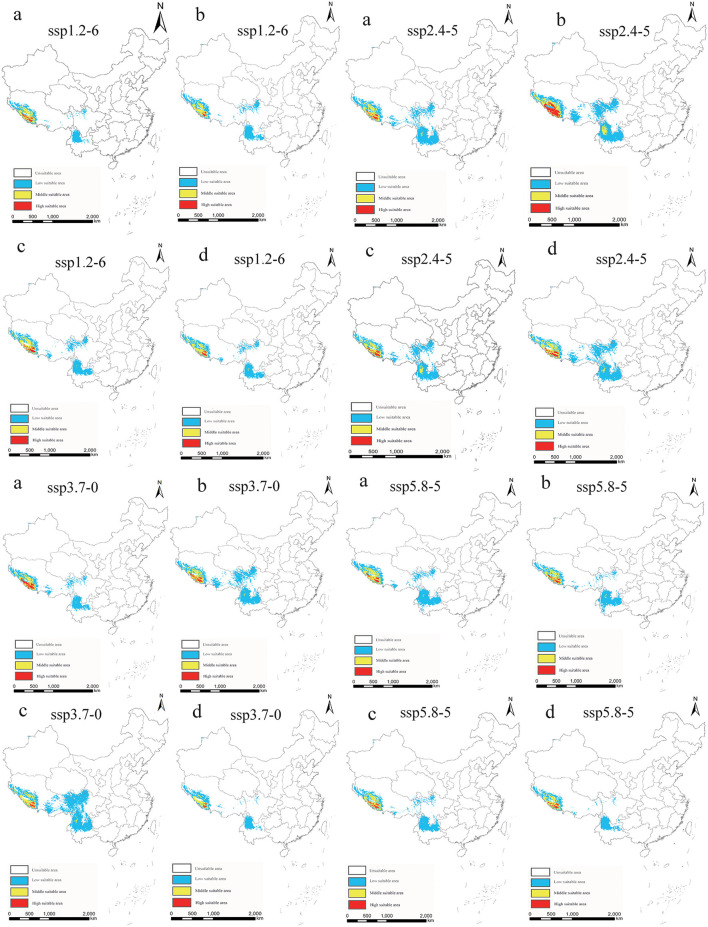
Suitable areas in China for *O. sexdentatus* under different future climate scenario models (a: 2021–2040, b: 2041–2060, c: 2061–2080, and d: 2081–2100).

**Figure 8:**
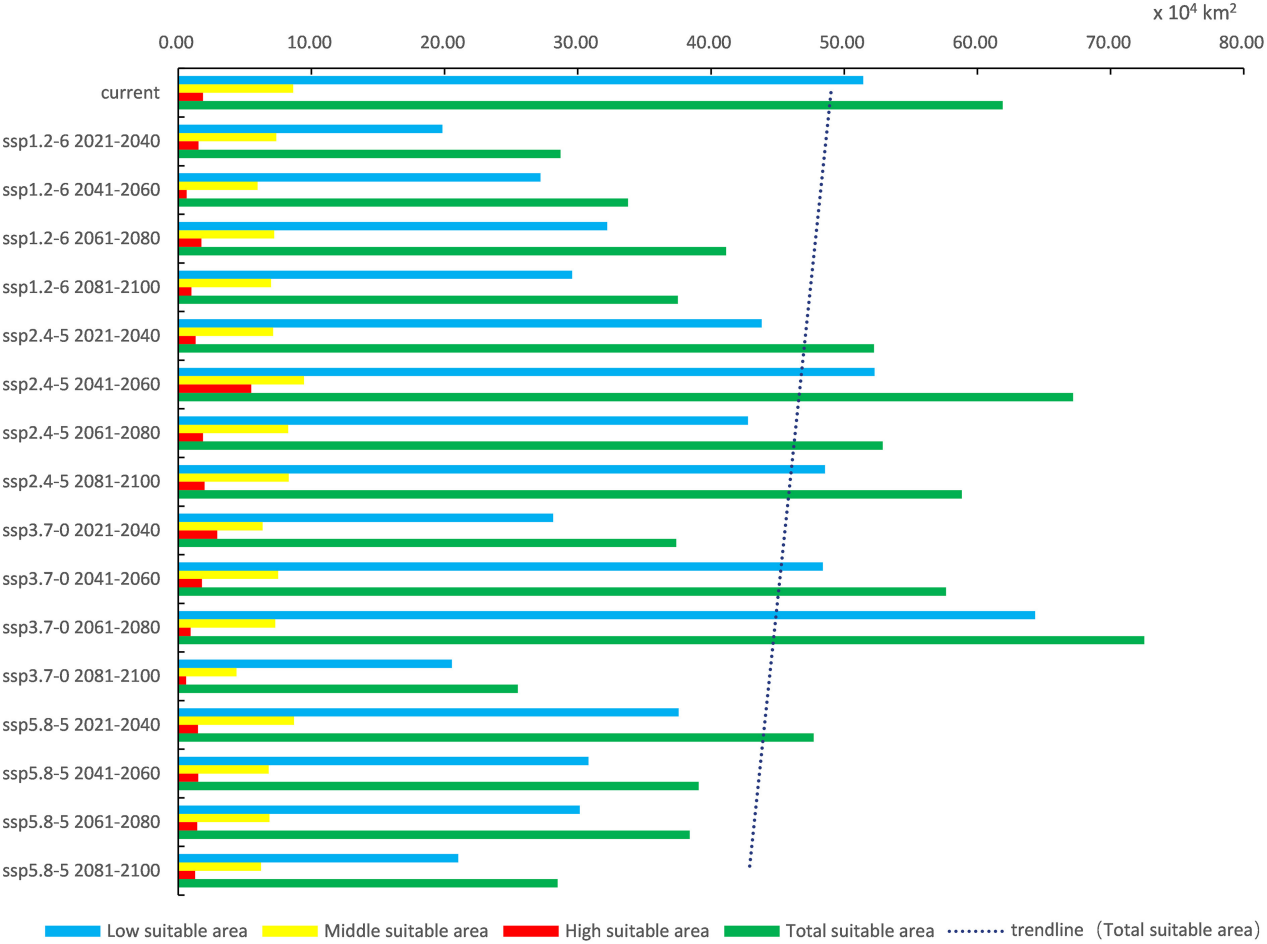
Variation trend of suitable areas for *O. sexdentatus* in China under current and future climate scenarios.

In light of the predictions for different future climate scenario models, the middle and high suitable areas for *O. sexdentatus* in China are distributed in southern Tibet and the northwestern part of Yunnan, and the low suitable areas are mainly distributed in Yunnan, Sichuan, and Tibet (Figure 7). As shown in Table 3, under the ssp3.7-0 climate scenario model from 2061 to 2080, *O. sexdentatus* has the largest suitable area in China (72.54 × 10^4^ km^2^). The overall trend of the suitable area in China is decreasing (Figure 8).

**Table 3:** Suitable areas of *O. sexdentatus* in China under current and future climate scenarios ( × 10^4^ km^2^).

**Climate scenarios**	**Period**	**Low suitable**	**Middle suitable**	**High suitable**	**Total area**	**Area change**	**Area change ratio (%)**
Current	1970–2000	51.43	8.62	1.86	61.91	0.00	
ssp1.2–6	2021–2040	19.83	7.36	1.52	28.71	−33.20	−53.63
	2041–2060	27.20	5.95	0.63	33.78	−28.13	−45.44
	2061–2080	32.20	7.20	1.74	41.14	−20.77	−33.55
	2081–2100	29.58	6.96	0.98	37.52	−24.39	−39.40
ssp2.4–5	2021–2040	43.82	7.13	1.30	52.25	−9.66	−15.60
	2041–2060	52.29	9.44	5.47	67.20	5.29	8.54
	2061–2080	42.78	8.25	1.86	52.89	−9.02	−14.57
	2081–2100	48.56	8.29	1.98	58.83	−3.08	−4.97
ssp3.7–0	2021–2040	28.15	6.33	2.92	37.40	−24.51	−39.59
	2041–2060	48.40	7.49	1.77	57.66	−4.25	−6.86
	2061–2080	64.34	7.28	0.92	72.54	10.63	17.17
	2081–2100	20.54	4.37	0.58	25.49	−36.42	−58.83
ssp5.8–5	2021–2040	37.57	8.68	1.47	47.72	−14.19	−22.92
	2041–2060	30.80	6.78	1.49	39.07	−22.84	−36.89
	2061–2080	30.14	6.85	1.41	38.40	−23.51	−37.97
	2081–2100	21.02	6.21	1.25	28.48	−33.43	−54.00

### Evaluation of the effect of the MaxEnt

**Figure 9:**
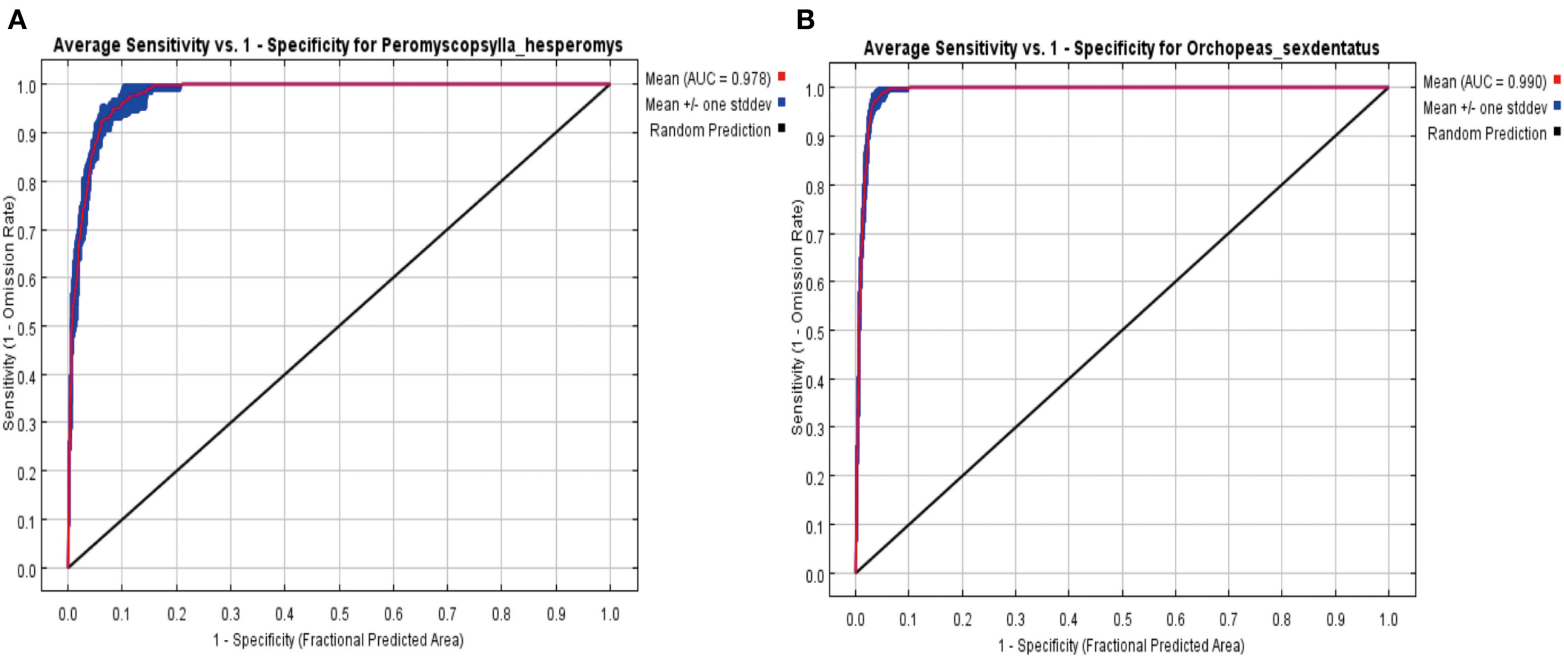
Receiver operating characteristic (ROC) curve by the MaxEnt for *P. hesperomys* **(A)** and *O. sexdentatus* **(B)**.

We evaluated the model's overall performance using a ROC curve (Figure 9). The accuracy of the predicted results was estimated by the AUC value. The AUC value of the MaxEnt model for *P. hesperomys* and *O. sexdentatus* s was 0.978 and 0.990, which revealed that the accuracy of the models was high.

### Risk assessment results

According to the constructed multi-indicator invasion risk assessment system and scoring criteria of each indicator, the comprehensive risk value of two representative U.S. plague vector fleas for invading China was calculated (Supplementary Table S2). The R-value of *P. hesperomys* and *O. sexdentatus* was 0.399 and 0.393, respectively, indicating that the risk levels of them invading China are moderate.

## Discussion

We used the MaxEnt model with optimized parameters to predict the suitable areas of plague vector fleas within China in the future under four SSPs scenarios, which served as an early warning. It provides the basis for preventing the plague vector fleas from invading China and developing relevant custom inspection and quarantine policies.

In the context of climate change, the total suitable area for *P. hesperomys* in China showed an overall trend of expansion, which may be due to global warming. The suitable areas of *P. hesperomys* in different periods under each climate scenario were higher than those under the current climate scenario. The large scale of suitable areas that appeared in China suggests that the prevention and control of *P. hesperomys* should be promoted. During the 2081–2100 period of the ssp1.2-6 climate scenario, the main range of suitable zones of *P. hesperomys* in China will extend from the Yellow River basin provinces to Zhejiang, Anhui, Jiangsu, Hubei, Chongqing, and a little in Guizhou, Hunan, Jiangxi, and Fujian. Increased monitoring of fleas in these areas is recommended to lower the risk of invasion and colonization. Our study found that suitable areas of *P. hesperomys* and *O. sexdentatus* in China include Yunnan, Sichuan, Chongqing, Tibet, Qinghai, Inner Mongolia, Guizhou, Xinjiang, Hebei, Gansu, Shaanxi, and Liaoning, which overlap with the distribution of their hosts (i.e., small rodents and woodrats) (28). Therefore, the risks of invasion, colonization, and the spread of the plague vector flea in these areas of China are relatively high.

According to the Köppen climate classification, the climate type of *P. hesperomys* distribution zones in the southeastern U.S. is the same as that of southern China for a temperate climate with hot summers but without any dry seasons (Cfa). Its climate is characterized by a minimum monthly mean temperature in the range of 0 to 18°C and a maximum monthly mean temperature of 22°C or higher within 1 year and high humidity. The Cfa has shown a significant expanding trend during the past 40 years in China (29), which is consistent with the results of our study: the suitable areas for *P. hesperomys* have a tendency to expand in southern China in the future. The expansion of the suitable area implies a rising public health risk for the population in the area, so it is necessary to strengthen health education on flea prevention and control of the population in the potentially suitable areas. In addition, the irregular geographical distribution of precipitation and high occurrences of extreme weather might have an impact on the distribution patterns for *P. hesperomys*.

### Major environmental factors which influence vector flea's survival

The efficiency of plague vector transmission for fleas is influenced by temperature, precipitation, and relative humidity (30, 31). Temperature is the primary factor influencing the growth of the plague vector flea, by accelerating flea growth observed at both larvae and pupae stages at higher temperatures (32). Therefore, global warming is conducive to the survival and activity of the plague vector flea. The main environmental variables influencing the distribution of potentially suitable areas for *P. hesperomys* were found to be Tmin11 (minimum temperature in November) and Tmin1 (minimum temperature in January), while the main environmental variables affecting the distribution of potentially suitable areas for *O. sexdentatus* were Bio3 (isothermality) and Bio2 (mean diurnal range). It also illustrated that high temperatures had a dramatic effect on the survival of vector fleas. Furthermore, a longer survival time of fleas was found at elevated humidity conditions. With rising humidity, the density of the host increases, and fleas may be more effective in transmitting *Yersinia pestis* (33). As the Cfa is characterized by high temperatures and humidity, the expansion of Cfa in southern China will increase the suitability of plague vector fleas as well as the risk of colonization and the ability to spread the plague.

### Invasion risk assessment and control measure of vector flea

In the background of climate change and global warming, originally unsuitable areas for biological survival are gradually transformed into suitable areas for survival, which lays the foundation for the invasion of alien species. The phenomenon of biological invasion becomes more frequent and serious (9). At present, invasive species have been discovered in all provincial-level administrative regions of China, and the southwest and southeast coasts are severe hazard areas (34).

With the implementation of China's strategies “The Silk Road Economic Belt” and “21st-Century Maritime Silk Road,” the risk of biological invasion in China continued to ascend. Our study revealed that the provinces along the Silk Road Economic Belt: Xinjiang, Gansu, Shaanxi, Chongqing, Sichuan, Inner Mongolia, Henan, Hebei, Qinghai, Beijing, and Tianjin were distributed with suitable areas for the plague vector flea. Furthermore, there are widespread suitable areas for vector fleas in Europe and Central Asia along the Silk Road. Thus, China Railway Express along the Silk Road Economic Belt offers a possible path for fleas to invade China. The third plague pandemic was spread by sea from India to the United States. The 21st-Century Maritime Silk Road starts in southern Europe and ends at the China port cities of Beihai, Haikou, Guangzhou, and Zhanjiang. Therefore, vector fleas invading China *via* shipping is possible.

The invasion risk assessment system reveals that two representative U.S. plague vector fleas are at moderate risk for invading China. Owing to the two species' significant potential for invasive damage in China, it is essential to bolster monitoring initiatives to prevent entry, colonization, and future spreading. We advise taking the following actions to prevent and control the vector flea: First, regulations control: departments for specific plague control in middle or high suitable areas and harbor cities with low suitable areas (i.e., Beijing, Tianjin, Qingdao, Rizhao, and Lianyungang) are needed to prevent the introduction of rodents and fleas. Second, use an early warning platform to master the dynamics of flea population. Flea surveillance has to be upped, especially in locations that are moderately or highly suitable for the two fleas. Third, environmental control: (1) hang sticky papers in the room to capture fleas, and (2) vacuum rooms infested with fleas, which can reduce 60% of eggs, 27% of larvae, and 95% of adults. Finally, chemical control: (1) clothing against fleas: spray 0.1% of dichlorvos on the bedding, and (2) to kill fleas on pigs, dogs, cats, and apply 1% permethrin or trichlorfon to the animal's body.

### The innovations and limitations

Innovations: First, there is no study on the risk of vector fleas invading China at present. This research provides a systematic research method for the visual screening of the key invasive vector fleas and their invasion risks in China. Second, this study found many potentially suitable areas for fleas with the risk of invading China. Although they have not been intercepted and discovered in China at present, they may still colonize and localize under certain conditions. Finally, the suitable areas for vector fleas are gradually expanding to southern China with global warming and the dramatic increase of the Cfa climate in China.

### Limitations

Constrained by historical climate data (1970–2000), there is a lack of the latest climate data to estimate current suitability zones, so there are limitations in this study. The MaxEnt model used in this study only considered the environmental adaptability of the species and only fitted selected environmental variables, such as temperature, precipitation, and elevation. A more accurate estimation of distribution in suitable areas requires additional variables such as topographic factors, soil moisture, interspecific relationships, vegetation cover type, population density, host density, land use, and economic indicators like GDP per capita. While this invasion risk assessment system involves a large number of related indicators, it still cannot fully and objectively represent the actual invasion risk of the species. Therefore, further optimization in the construction and screen of the indicator system is required.

## Conclusion

From a worldwide perspective, our research offered scientific data about the distribution of the suitable areas of *P. hesperomys* and *O. sexdentatus*. Both species have large potential suitable areas in China, the temperature has a vital impact on the distribution of suitable areas, and the suitable areas of *P. hesperomys* are expanding into the southern part of China under global warming. The invasion risk assessment system was adopted, and both species were found to be of moderate invasion risk for China, which served as an early warning for biological invasion in China. China should develop scientific prevention and control strategies and strengthen surveillance of its related alarm systems to prevent the invasion of overseas flea vectors.

## Data availability statement

The original contributions presented in the study are included in the article/Supplementary material, further inquiries can be directed to the corresponding authors.

## Author contributions

HH and QL: conception, design of the research, and critical revision of the manuscript for intellectual content. HL, YL, LD, CL, LZ, BW, DM, QM, and JW: literature review and acquisition of data. HL: analysis and interpretation of the data and writing of the manuscript. All authors read and approved the final draft.

## Supplementary material

Supplementary Table 1 Invasion risk assessment system for vector biology and reference standard of index assignment.

Supplementary Table 2 Two vector fleas risk assessment indicator score.

Supplementary Table 3 Occurrence points of *Peromyscopsylla hesperomys*.

Supplementary Table 4 Occurrence points of *Orchopeas sexdentatus*.
