# Supplementary material for: Predicting global potential distribution of Peromyscopsylla hesperomys and Orchopeas sexdentatus and risk assessment for invading China under climate change
Source: Front Public Health. 2023 Jan 5;10:1018327. doi: 10.3389/fpubh.2022.1018327 (PMC9850084; doi:10.3389/fpubh.2022.1018327)
Supplement: Supplementary Table 1 — Invasion risk assessment system for vector biology and reference standard of index assignment. [file Table_1.DOCX]

| Table S1 Invasion risk assessment system for vector biology and reference standard of index assignment. | | | | | | | |
| --- | --- | --- | --- | --- | --- | --- | --- |
| Tier 1 Indicators | Tier 2 Indicators | Tier 3 Indicators | Scoring Criteria | | | | |
|  |  |  | 0 | 0＜x≤0.25 | 0.25＜x≤0.5 | 0.5＜x≤0.75 | 0.75＜x≤1 |
| 1.Entry possibility(*P*_1_) | 1.1 Entry paths | 1.1.1 International Distribution | / | No invasion reported from abroad | Invaded to two continents outside the continent of origin | Invaded countries bordering China | Has invaded China |
|  |  | 1.1.2 The situation of trade and human contact with invasion nation in the countries or regions of the current distribution of the species | No trade or people traffic | Low | Middle | Frequent | Very Frequent |
|  |  | 1.1.3 Biological Invasion probability by nature | Cannot naturally import | / | / | / | Can be introduced actively or by natural |
|  | 1.2 Transport risks and quarantine efficiency | 1.2.1 Frequency of intercepting | No interception record | Intercepted annually | Intercepted quarterly | Intercepted monthly | Intercepted weekly |
|  |  | 1.2.2 Quarantine efficiency ^a^ | Very effective | Effective | Moderate | Low | Very low |
| 2.Colonization and spread possibility(*P*_2_) | 2.1 Reproductive capacity | 2.1.1 Reproduction generation |  | One generation per year | Two generation per year | Three-four generations per year | More than four generations per year |
|  |  | 2.1.2 Reproduction mode | / | Reproduce without parthenogenesis | / | / | Reproduce with parthenogenesis |
|  |  | 2.1.3 Maximum fecundity of one female | Reproduction  quantity＜10 | 10＜Reproduction  quantity≤50 | 50＜Reproduction  quantity≤100 | 100＜Reproduction  quantity≤200 | Reproduction quantity>200 |
|  | 2.2 Suitability | 2.2.1 Proportion of suitable areas  in invading countries (%) | 0 | 0.01%＜Proportion≤1% | 1%＜Proportion≤5% | 5%＜Proportion≤10% | 10%＜Proportion≤1 |
|  |  | 2.2.2 Maximum suitable probability in invading countries | Probability≤0.01 | 0.01＜Probability≤0.1 | 0.1＜Probability≤0.2 | 0.2＜Probability≤0.5 | 0.5＜Probability≤1 |
|  |  | 2.2.3 Area of invading countries suitable(×10^4^km^2^) | 0 | 0＜Suitable area≤20 | 20＜Suitable area≤50 | 50＜Suitable area≤100 | 100＜Suitable area |
|  | 2.3 Resistant ability | 2.3.1 Resistant ability^b^ | None | Weak | Moderate | Strong | Extremely strong |
|  | 2.4 Diffusion capacity | 2.4.1 Diffusion capacities by transportation | None | Weak | Moderate | Strong | Extremely strong |
|  |  | 2.4.2 Ability of active flight and disperse by  natural vectors | None | Weak | Moderate | Strong | Extremely strong |
|  | 2.5 Control effect | 2.5.1 Controlling effect of biological factors | / | Natural enemies widely distributed in suitable area | Natural enemies less distributed in suitable area | Natural enemies very rarely distributed in suitable area | Natural enemies almost absent in suitable area |
|  |  | 2.5.2 Human management and monitoring effect | Very effective | Effective | Moderate | Low | Very low |
|  |  | 2.5.3 Eradication and control difficulty | Very easy | easy | Moderate | difficulty | Very difficulty |
| 3. Damage consequences(*P*_3_) | 3.1 Ecological Hazards | 3.1.1 Impact on soil, air and surface | Extremely Weak | Weak | Moderate | Strong | Extremely strong |
|  |  | 3.1.2 Impacts on native species and biodiversity | Extremely Weak | Weak | Moderate | Strong | Extremely strong |
|  |  | 3.1.3 The ability to carry other pathogenic microorganisms | Extremely Weak | Weak | Moderate | Strong | Extremely strong |
|  | 3.2 Social Hazards | 3.2.1 Impact on the life and work of residents | Extremely Weak | Weak | Moderate | Strong | Extremely strong |
|  |  | 3.2.2 Economic loss to relate industries | Extremely Weak | Weak | Moderate | Strong | Extremely strong |
|  |  | 3.2.3 Economic loss due to health hazards to the population | Extremely Weak | Weak | Moderate | Strong | Extremely strong |
|  |  | 3.2.4 Economic losses to other sides | Extremely Weak | Weak | Moderate | Strong | Extremely strong |

a: Difficulty of quarantine discovery for invasive species

b: Resilience to adverse environments
