# Supplementary material for: Predicting global potential distribution of Peromyscopsylla hesperomys and Orchopeas sexdentatus and risk assessment for invading China under climate change
Source: Front Public Health. 2023 Jan 5;10:1018327. doi: 10.3389/fpubh.2022.1018327 (PMC9850084; doi:10.3389/fpubh.2022.1018327)
Supplement: Supplementary Table 2 — Two vector fleas risk assessment indicator score. [file Table_2.DOCX]

| Table S2. The two fleas vector risk assessment indicator score | | |
| --- | --- | --- |
|  |  |  |
| Indicators score | *P. hesperomys* | *O. sexdentatus* |
| *P*_111_ | 0.25 | 0.25 |
| *P*_112_ | 1 | 1 |
| *P*_113_ | 0 | 0 |
| *P*_121_ | 0 | 0 |
| *P*_122_ | 0.25 | 0.25 |
| *P*_211_ | 0.5 | 0.5 |
| *P*_212_ | 0.25 | 0.25 |
| *P*_213_ | 0.75 | 0.75 |
| *P*_221_ | 1 | 0.75 |
| *P*_222_ | 1 | 1 |
| *P*_223_ | 1 | 0.75 |
| *P*_231_ | 0.75 | 0.75 |
| *P*_241_ | 0.5 | 0.5 |
| *P*_242_ | 0.25 | 0.25 |
| *P*_251_ | 0.25 | 0.25 |
| *P*_252_ | 0.5 | 0.5 |
| *P*_253_ | 0.25 | 0.25 |
| *P*_311_ | 0.25 | 0.25 |
| *P*_312_ | 0.25 | 0.25 |
| *P*_313_ | 0.75 | 0.75 |
| *P*_321_ | 0.5 | 0.5 |
| *P*_322_ | 0.5 | 0.5 |
| *P*_323_ | 0.75 | 0.75 |
| *P*_324_ | 0.5 | 0.5 |
